# Physiology-Based Pharmacokinetic Study on 18β-Glycyrrhetic Acid Mono-Glucuronide (GAMG) Prior to Glycyrrhizin in Rats

**DOI:** 10.3390/molecules27144657

**Published:** 2022-07-21

**Authors:** Mengxin Cao, Jiawei Zuo, Jian-Guo Yang, Chenyao Wu, Yongan Yang, Wenjian Tang, Lili Zhu

**Affiliations:** 1 Anhui Province Key Laboratory of Major Autoimmune Diseases, School of Pharmacy, Anhui Medical University, Hefei 230032, China; caomengxin0711@163.com (M.C.); zuojiawei2022@163.com (J.Z.); yangjgyx@163.com (J.-G.Y.); chengyao.wu@hotmail.com (C.W.); 2 Huainan Municipal Food and Drug Inspection Center, Huainan 232000, China; 3 Jiangsu Nature Biological Engineering Technology Co., Ltd., Nanjing 210023, China; yangyan73@163.com

**Keywords:** GAMG, glycyrrhetic acid, glycyrrhizin, pharmacokinetics, pharmacodynamics, tissue distribution

## Abstract

To understand that 18β-Glycyrrhetic acid 3-O-mono-*β*-D-glucuronide (GAMG) showed better pharmacological activity and drug-like properties than 18β-Glycyrrhizin (GL); a rapid and sensitive HPLC-MS/MS method was established for the simultaneous determination of GAMG and its metabolite 18β-Glycyrrhetinic acid (GA) in rat plasma and tissues after oral administration of GAMG or GL. This analytical method was validated by linearity, LLOQ, specificity, recovery rate, matrix effect, etc. After oral administration, GAMG exhibited excellent *C*_max_ (2377.57 ng/mL), *T*_max_ (5 min) and AUC_0-T_ (6625.54 mg/L*h), which was much higher than the *C*_max_ (346.03 ng/mL), *T*_max_ (2.00 h) and AUC_0-T_ (459.32 mg/L*h) of GL. Moreover, GAMG had wider and higher tissue distribution in the kidney, spleen, live, lung, brain, etc. These results indicated that oral GAMG can be rapidly and efficiently absorbed and be widely distributed in tissues to exert stronger and multiple pharmacological activities. This provided a physiological basis for guiding the pharmacodynamic study and clinical applications of GAMG.

## 1. Introduction

Glycyrrhizin (GL) is the major bioactive component in licorice with diverse pharmacological activities [1,2]. 18β-Glycyrrhetic acid 3-O-mono-*β*-D-glucuronide (GAMG), a triterpene glycoside containing one molecule of 18β-H-oleanane-type aglycone and one molecule of glucuronic acid, can be observed via enzymolysis or metabolism by elimination of the distal glucuronic acid [3,4]. GAMG exhibited stronger anticancer action through down-regulating the expression of protein p65 [5], improved CCl_4_-induced hepatic fibrosis by suppressing NF-κB and MAPK signaling pathway [6], and alleviated single-walled carbon nanotubes-induced lung inflammation and fibrosis in mice by the PI3K/AKT/NF-κB signaling pathway [7]. Compared to GL, GAMG showed stronger physiological activities than GL [5,6,7,8,9]. GAMG has been developed as a natural high-potency sweetener due to its higher solubility and better taste [9,10]. Although GAMG possessed appropriate chemical polarity for drug development, the physiology-based pharmacokinetic study would prompt us to reveal why GAMG showed stronger physiological activity than GL.

**Figure 1:**
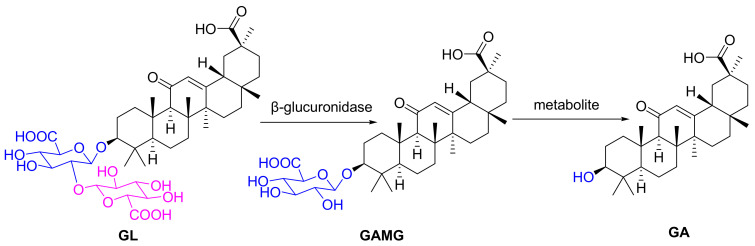
The chemical structural formulas of GL, GAMG and GA.

GL was metabolized by human intestinal microflora to two active products, namely a main 18β-glycyrrhetinic acid (GA) and a minor GAMG (Figure 1). After intravenous administration of GL, both GAMG and GA were produced in the liver, and GAMG can be excreted into the intestine through bile, and then be further metabolized into GA by intestinal flora and absorbed into the blood, which suggested enterohepatic circulation in the metabolism of GL [11,12]. GL can affect the metabolism of glucuronyltransferase and CYPs in the liver [13,14,15,16]. The precise glycosylation of C3-OH mediated by microbial glycosyltransferase has been used to efficiently biosynthesize GAMG by transferring a glucuronosyl moiety [17,18]. Recently, an increasing number of physical, chemical, and biotechnological approaches were used for the precise extraction and synthesis of GAMG [4,19,20,21]. GAMG has great potential for new drug development due to better solubility and more stable chemical properties than GL.

As the active metabolite of GL, GAMG has attracted considerable attention, especially in the food and pharmaceutical industries, due to its natural sweetness and wide biological activities [8,9,22]. Until now, there are no reports on pharmacokinetics of GAMG; therefore, it is necessary to investigate the bioavailability, tissue distribution and disposition of GAMG in vivo. In this work, an LC-MS/MS method with high specificity and high sensitivity was established to detect GAMG and GA in rat plasma and tissues at the same time. It is of great significance to understand the pharmacokinetics and target organ dosimetry properties of GAMG and large molecular biologics for disease treatments [23,24]. This work will help to elucidate the better pharmacological activity of GAMG by comparing the pharmacokinetic parameters after the same dose of GL and GAMG by intragastric administration.

## 2. Materials and Methods

### 2.1. Materials

GAMG, GA, and internal standard 18β-glycyrrhetinic acid 30-(*N*-2-hydroxylethyl)amide (IS) were provided by the School of pharmacy, Anhui Medical University. Methanol and formic acid were HPLC grade and purchased from Fisher (Hampton, NH, USA) and Aladdin (Shanghai, China), respectively. The experimental water was distilled water (Watson, Hong Kong). Other chemicals were HPLC or analytical grade.

### 2.2. HPLC-MS/MS Conditions

Quantification of GAMG in the biological samples was performed using an Agilent 1200 series high-performance liquid chromatography with tandem Agilent 6460 triple quadrupole mass spectrometer (Agilent Technologies, Santa Clara, CA, USA). An RRHD Eclipse Plus C18 column (50 mm × 2.1 mm, i.d., 1.8 μm) was employed to separate GAMG and GA from complex biomatrices. Injection volume was 10 μL. Elution was fulfilled with an isocratic mobile phase (delivered at 0.2 mL/min) consisting of methanol (0.1% formic acid)-10% methanol (85:15, *v*/*v*). The autosampler was maintained at 4 °C. GAMG and internal standard (IS) were quantified via a positive electrospray ionization interface. They were monitored by multiple reaction monitoring (MRM) mode with transitions of *m*/*z* 647.4 → 453.5 for GAMG, *m*/*z* 471.3 → 189.3 for GA and *m*/*z* 514.4 → 189.4 for IS. The compound-dependent parameters, such as collision energy and fragmentor voltage, were optimized as 15 eV and 148 V for GAMG, 41 eV and 144 V for GA and 32 eV and 90 V for IS, respectively. High purity nitrogen served as both the nebulizing and drying gases. Other parameters of the mass spectrometer were also optimized and set, including spray voltage (3.2 kV), ion source temperature (340 °C), nebulizer pressure (30 psi), and capillary temperature (270 °C).

### 2.3. Preparation of Calibration Standard QC Samples

GAMG and GA were dissolved in methanol at a concentration of 100 µg/mL as stock solutions. A mixed stock solution was prepared by mixing the two stock solutions to yield the following final concentrations: 3000, 2000, 1000, 500, 200, 100, 50, 20, 10, and 5 ng/mL. Four concentrations of QC samples, including the lower limit of quantification (1 ng/mL, 3 ng/mL, 40 ng/mL, 250 ng/mL for GAMG and 0.5 ng/mL, 1.5 ng/mL, 20 ng/mL, 120 ng/mL for GA), were also prepared using the same method. Moreover, IS solution was diluted to a final concentration of 500 ng/mL. Serial dilution calibrations were prepared by spiking the appropriate working solution into blank plasma or different blank tissue homogenates. All solutions were stored at 4 °C.

### 2.4. Plasma and Tissue Sample Processing Methods

All biological samples were frozen at −40 °C. Samples were removed and thawed at ambient temperature before analysis. After thawing, they were processed by simple and effective methanol precipitation of proteins.

First, 100 μL of plasma sample (or tissue homogenate) was precisely added to a 1.5 mL polypropylene (PP) tube. Then, 10 μL methanol and 10 μL IS solution (500 ng/mL) were added. Subsequently, 300 μL methanol was added to initiate simple protein precipitation in the sample. The mixture was vigorously vortexed for 30 s, followed by centrifugation at 4000 rpm for 10 min at −4 °C. Finally, 270 μL of the supernatant was aspirated quantitatively and filtered with a 0.22 μm organic filter membrane. Take 10 μL of the filtrate for LC-MS/MS analysis.

### 2.5. Methodology Validation

#### 2.5.1. Specificity

Specificity was demonstrated by analyzing blank biosamples, spiked biological samples, and actual biosamples following intragastric administration of GAMG to exclude the influences of endogenous interferences. There should be no endogenous responses at the retention time of GAMG, GA and IS in blank biological samples.

#### 2.5.2. Linearity and Lower Limits of Quantification (LLOQ)

Using weighted (1/x^2^) linear regression, a calibration curve was generated from the peak area ratios of analyte and IS to the corresponding concentration of analyte, the coefficient (r) of which should be above 0.99. The limit of detection (LOD), depicted as the concentration of analyte giving a signal-to-noise ratio of 3. LLOQ, depicted as the concentration of analyte giving an S/N ratio above 10, could be evaluated by six replicate analyses with a percentage relative standard deviation (precision) and a percentage relative error (accuracy) not exceeding ±20%.

#### 2.5.3. Accuracy and Precision

For intra-assay variations, three QC samples with low, medium, and high levels (*n* = 6) were determined on the same day. For inter-assay variations, three QC samples with low, medium, and high levels (*n* = 6) were analyzed on three consecutive days. Precision is expressed as RSD% (relative standard deviation), while accuracy is expressed as RE% (deviation from the true value). The average value of RSD and RE was required to be within ±15%, which shows that the precision and accuracy of intra-assay and inter-assay variation was favorable.

#### 2.5.4. Recovery and Matrix Effect

Three QC samples with low, medium, and high levels (*n* = 6) were analyzed to assess the recovery and matrix effects of GAMG and GA in various biological samples. The extraction recovery for analyte was estimated by comparing the peak response of extracted QC samples with those of the spiked post-extraction blank biological matrix at equivalent levels. The matrix effect was carried out by comparing the mean peak responses of the analyte in the spiked samples (post-extraction) with those of the analyte contained in the standard solutions at the corresponding three levels.

#### 2.5.5. Stability

Stability measurements were conducted to assess the stability of biological samples exposed to different storage and processing conditions. For each condition, three levels of QC samples (*n* = 3) were analyzed. Short-term stability was tested after the exposure of QC samples at room temperature for 4 h. Long-term stability was determined by storing the QC samples at −40 °C for one month. QC samples that underwent three freeze-thaw cycles (from −40 °C to room temperature) were investigated for freeze-thaw cycle stability. An acceptance criterion for all stability samples did not exceed ±15%.

#### 2.5.6. Dilution Reliability

Two drug-containing plasma samples (*n* = 5) were prepared with a high concentration of GAMG and GA, diluted with blank plasma, processed according to the method of plasma sample processing, and measured by sample injection. The measured value of concentration multiplied by the dilution factor is compared with the theoretical value, and the results of RE% and RSD% should not exceed ±15%.

### 2.6. Animal Experiments and Sampling

Sixty Sprague-Dawley (SD) rats, including 48 males and 12 females (weight 180–220 g), were purchased from Jinan Pengyue Experimental Animal Breeding Co., Ltd. (Jinan, China). The rats were housed in an air-conditioned laboratory (18–22 °C, 40–60% humidity) with a 12-h dark/light cycle and acclimatized under the above conditions for 7 days. In addition, all experimental rats were starved overnight to eliminate dietary interference, but they were allowed to drink freely before dosing.

For pharmacokinetics studies, 24 SD rats were stochastically assigned to three groups (eight rats per group, half males and half females). The low, medium, and high dose groups were intragastric administered GAMG at 7.5, 15 and 30 mg/kg, and the high dose group was recorded as the GAMG group. The remaining six male rats were recorded as the GL group by intragastric administration of 30 mg/kg GL. Following intragastric administration, 300 μL of blood samples from eye postorbital venin were collected in sodium heparin-coated vials at the designated time points (0 (before administration), 5, 15, 30, 45 min and 1, 2, 4, 8, 12, 14, 24, 36, 48 h) and then immediately centrifuged (−4 °C, 4000 rpm, 10 min). Plasma (100 μL) was harvested into a new centrifuge tube and frozen (−40 °C) before use.

For the tissue distribution studies, 30 SD rats were assigned to five groups at random (*n* = 6 each group). The first group of rats was euthanized by decapitation to obtain blank tissue samples prior to administration. The other four groups were sacrificed at 0.5, 1, 4, and 12 h time points following intragastric dosing of GAMG at 30 mg/kg. Afterwards, tissue samples, including heart, liver, spleen, lung, kidney, brain, and small intestine, were immediately excised and flushed with sodium chloride solution (0.9%) to remove the impact of blood or chyme. Subsequently, all tissues were wiped dry with blotting paper and kept at −40 °C.

### 2.7. Statistical Analysis

Using the drug and statistics 2.0 program (Chinese Pharmacological Society) software, the PK parameters of each component were obtained through the non-compartmental model and statistical moment parameters. The area under the plasma concentration-time curve from 0 to the last time (AUC_0-t_), elimination half-life (*t*_1/2_), clearance (*CL*), mean residence time (MRT_0-t_) and apparent volume of distribution (*V*_d_) were calculated, while the peak concentration (*C*_max_) and time-to-peak concentration (*T*_max_) were obtained directly from each individual set of data. SPSS 17.0 software was used to analyze each component, and PK parameters were analyzed by *t* tests. All values are presented as the mean ± standard deviation. Differences were considered statistically significant at *p* < 0.05.

## 3. Results and Discussion

### 3.1. Chromatographic and Mass Spectrometric Condition Optimization

Adjusting the mobile phase in different proportions, it was found that the peak response and peak shape of each test substance are the best under the ratio of methanol (0.1% formic acid) to 10% methanol (85: 15, *v*/*v*). Formic acid (0.1%) can effectively improve the peak shape, reduce the tailing effect, and improve sensitivity.

In this experiment, the positive-ion scan mode was used. In the MS scan, the capillary outlet voltage (Fragmentor) of the instrument was adjusted, and the most abundant precursor ion was found under the appropriate fragmentation voltage. The main precursor ions obtained for GAMG, GA and IS were at *m*/*z* 647.4, *m*/*z* 471.3 and *m*/*z* 514.4, respectively. Then, a product ion scan of the main precursor ion was selectively performed, and the collision energy (CE) was optimized to make the quantitative product ion the highest abundance. The highest peak response was used as the quantitative product ion, and the second height peak response was used as the qualitative product ion (Figure S1). The optimized mass spectrometer parameters of each ion pair are shown in Section 2.2.

For selecting the ideal internal standard, a similar chemical behavior and a suitable retention time were of significant importance. 18β-Glycyrrhetinic acid 30-(*N*-2-hydroxylethyl)amide was selected as IS because it was similar to the analyte in chemical structure, chromatographic behavior, MS characteristics and recovery. When mixed with GAMG and GA to prepare a mixed standard solution, it will not affect the determination of the other two substances.

### 3.2. Method Validation

#### 3.2.1. Specificity

Figure S2 illustrates typical chromatograms of GAMG, GA and IS derived from rat blank plasma samples, spiked plasma samples and actual plasma samples. It was found that IS, GAMG, and GA appeared in sequence within 5 min (retention time: 1.5 min for IS, 1.6 min for GAMG, 2.0 min for GA). Each analyte has strong specificity. Impurities in plasma do not affect the concentration determination of the substance to be tested.

#### 3.2.2. Calibration Curve and Linearity

A summary of calibration curves in all biological samples is listed in Table 1 and Table 2. All calibration curves displayed perfect linearity (*r* ≥ 0.99) over the corresponding linearity range. The LLOQs of GAMG and GA were 2 ng/mL and 0.5 ng/mL in plasma, and 10 ng/mL and 1 ng/mL in various organs, respectively. It can meet the requirements of sufficient sensitivity for biological analysis in vivo. The RSD and RE values of the LLOQ (*n* = 6) of each biological sample were within 20%.

#### 3.2.3. Accuracy and Precision

Table 3 summarizes intra-assay and inter-assay variations in GAMG and GA at three QC levels concentrations in plasma samples. The RSD values of precision ranged from 4.31% to 14.20% and 6.33% to 13.28%, respectively. The RE values of accuracy are between −5.02% and 14.75%. The above results were all in acceptable limits, indicating that the approach exhibited satisfactory reproducibility for quantification of the analyte in plasma samples.

#### 3.2.4. Recovery and Matrix Effect

The RSD values of the recoveries for GAMG and GA at three QC levels in plasma samples ranged from 7.58% to 12.71%, while IS ranged from 9.04% to 11.94%, all within ±15%. The coefficient of variation CV of the internal standard normalized matrix factor calculated for six batches of matrix is between 6.88% and 13.60%, less than 15%. The matrix effect between the analyte and IS meets the requirements, and the ion from biomatrices can be considered to have no influence on the content determination of the analyte (Table 4).

#### 3.2.5. Stability

The stability results in plasma samples suggested that GAMG, GA, and IS were stable under all the evaluated conditions, such as 25 °C for 4 h, −40 °C for one month, and three freeze-thaw cycles (Table 5). The RSD values of the stability for GAMG and GA at three QC levels in plasma samples were within ±15%. This shows that the analyte has good stability under the sampling environment and various storage conditions.

#### 3.2.6. Dilution Integrity

High-concentration QC samples of GAMG and GA (*n* = 5) were prepared, and the corresponding multiples were diluted with blank plasma for determination. The RE and RSD values obtained by multiplying the measured value by the dilution factor were both within ±15%, which revealed that plasma samples with concentrations exceeding the ULOQ could be reanalyzed after appropriate dilution with blank plasma (Table S1).

### 3.3. Pharmacokinetic Studies

#### 3.3.1. Dose Correlation and Gender Differences

**Figure 2:**
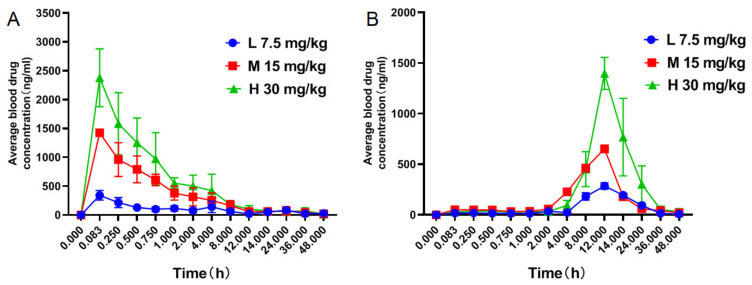
The average blood concentration-time curve of each analyte ((**A**): GAMG; (**B**): GA) at 0–48 h after ig low (L), medium (M) and high (H) three dose groups of GAMG (*n* = 8).

After intragastric administration of GAMG low, medium, and high dose groups, blood was taken at different time points to determine the average blood concentration-time curve of GAMG and GA in rats (Figure 2). After intragastric administration of GAMG at three doses of 7.5, 15 and 30 mg/kg, *C*_max_ and *T*_max_ of GAMG were 341.65 ng/mL and 0.083 h, 1425.15 ng/mL and 0.083 h, 2469.66 ng/mL and 0.083 h, respectively, while *C*_max_ and *T*_max_ of GA were 368.53 ng/mL and 10.25 h, 736.93 ng/mL and 11.00 h, 1428.70 ng/mL and 12.25 h, respectively. The main non-compartmental pharmacokinetic parameters of GAMG and GA were obtained from the DAS 2.0 data processing results (Tables S2 and S3).

After intragastric administration of GAMG at three doses of 7.5, 15, and 30 mg/kg, *C*_max_ and AUC_0-t_ showed dose correlations. As the dose increases, *C*_max_ and AUC_0-t_ also increase proportionally (Figures S3 and S4). The PK parameters of GAMG and its metabolite GA in rat plasma of the three dose groups were tested by Dunnett’s *t* test using SPSS 17.0 software, and the *p* values obtained were all >0.05 (Table 6 and Table 7). The PK parameters of GAMG and GA in the three dose groups were not significantly different by sex.

#### 3.3.2. Comparison of Pharmacokinetics between GAMG Group and GL Group

**Figure 3:**
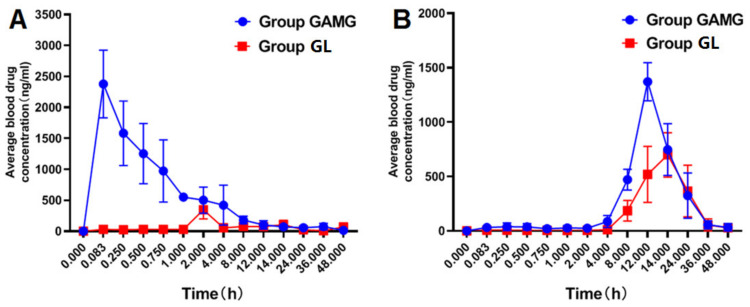
Average plasma concentration-time curve of each analyte ((**A**): GAMG; (**B**): GA) after intragastric administration in rats (*n* = 6).

After intragastric administration of GL and GAMG at the same dose (30 mg/kg), the average plasma concentration-time curves of GAMG and GA in the two groups of rats are shown in Figure 3. The two sets of PK parameter results obtained by DAS 2.0 software were significantly different (Table 8 and Table 9).

The results showed that the metabolite GAMG should have better absorption. After oral administration of GAMG, *C*_max_ and *T*_max_ of GAMG were 2377.57 ng/mL and 0.083 h, respectively, while after oral administration of GL, *C*_max_ and *T*_max_ of GAMG were 346.03 ng/mL and 2.00 h, respectively (Figure 2 and Figure 3). Obviously, oral GAMG could be directly and rapidly absorbed in the body, thus, maximum peak time of oral GAMG (*T*_max_ = 5 min) was much less than that of oral GL (*T*_max_ = 120 min). After oral GL, GL was first metabolized by intestinal microflora, then GAMG was absorbed [10,11]. The area under the drug-time curve of oral GAMG was much higher than that of oral GL. AUC_0-T_ of oral GAMG (6625.54 mg/L*h) was 14.4 times as that of oral GL (459.32 mg/L*h). The oral availability of GAMG was much higher than that of GL. Therefore, the rapid and efficient absorption of GAMG was one of cause of GAMG prior to GL.

Although GAMG was the active metabolite of GL, both GL and GAMG could be metabolized into GA, and further be transferred to GAMG through Phase II metabolism of conjugation with glucuronic acid. After oral administration of GAMG or GL, the maximum peak time of GA was significantly delayed, and the metabolite GA reached its peak at more than 12 h (Figure 2 and Figure 3). After oral GAMG, *C*_max_ and *T*_max_ of GA were 1412.58 ng/mL and 12.33 h, respectively, while after oral GL, *C*_max_ and *T*_max_ of GA were 747.08 ng/mL and 13.67 h, respectively, in which *C*_max_ of the former was almost twice as that of the latter. The area under the drug-time curve of GA of oral GAMG (AUC_0-T_ = 15,252.54 mg/L*h) was a little higher than that of oral GL (AUC_0-T_ = 11,598.49 mg/L*h). Interestingly, although oral GL needed to be metabolized via intestinal flora and transferred into GA through the enterohepatic circulation, the oral availability of GL and GAMG was close to the metabolite GA. Therefore, the higher concentration of metabolite GA of GAMG can also explain its better biological activity.

Although the metabolite GA of oral GAMG and GL reached its peak at 12 h, there was basically no accumulation in the body after 48 h (Figure S3), which implied that GAMG had the same benign safety as GL. Because GAMG had better anti-inflammatory activity than GA [6], oral medication of GAMG can produce faster and longer lasting pharmacological effects compared with GL. This explained that GAMG exhibited better antitumor and anti-inflammatory activity than GL [5,6,7,8,9,10]. Therefore, GAMG can be developed as a drug with multiple physiological functions.

The study on simultaneous determination of GL and GA in human plasma by LC–MS/MS reported the chronological changes in the concentration of GL and GA after oral administration of GL [25]. GA was barely detectable 4 h after ingestion of GL and its concentration rapidly increased by 6 h. The *C*_max_ of GA (200.3 ± 60.3 ng/mL) was ~8-fold higher than that of GL (24.8 ± 12.0 ng/mL). After reaching *T*_max_ (10.3 ± 2.7 h), GA levels gradually decreased with time. The AUC_0–t_ of GA was 3550.8 ± 470.2 ng h/mL. In the future, we will carry out the study on pharmacokinetics of GAMG.

### 3.4. Tissue Distribution Study

**Figure 4:**
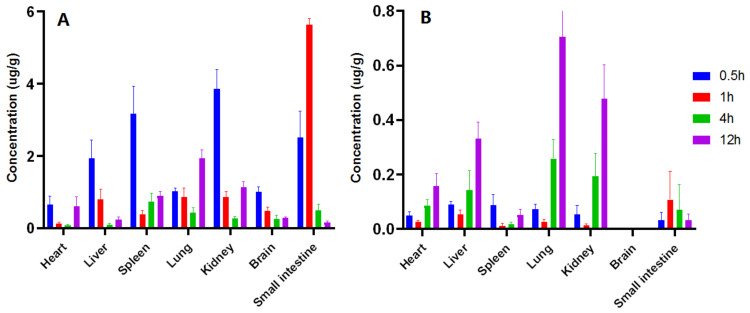
The concentration of metabolites GAMG (**A**) and GA (**B**) in various tissues at different time points.

Tissue distribution after intragastric administration of GAMG (30 mg/kg) was investigated by the validated HPLC-MS/MS method. The distribution concentration results of GAMG and GA were shown in all the tested tissues at 0.5, 1, 4, and 12 h (Table S4), and the tendency results were shown (Figure 4). It was found that the distribution of GAMG has obvious targets, with more distribution in kidney (3.859 μg/g), spleen (3.167 μg/g), small intestine (2.509 μg/g), liver (1.946 μg/g) and lung (1.035 μg/g); however, the distribution of GAMG in the heart (0.657 μg/g) and brain (1.012 μg/g) was observed at 0.5 h after administration, indicating that GAMG can pass through the blood-brain barrier. Until 12 h after administration, there were higher concentrations of GAMG in lung (1.942 μg/g), kidney (1.136 μg/g) and spleen (0.894 μg/g). The high concentration of GAMG in the small intestine conformed that GAMG, the metabolite of GL through intestinal flora, was more efficiently absorbed in the small intestine.

The distribution of metabolite GA in all tissues except brain tissues gradually increased from 0.5 h to 12 h after administration. The distribution of GA in the lung (0.259 μg/g), kidney (0.193 μg/g), and liver (0.142 μg/g) reached higher concentrations at 4h. Lung (0.705 μg/g), kidney (0.479 μg/g) and liver (0.332 μg/g) were close to their peaks at 12 h, but the concentrations in the brain were basically undetectable, indicating that GA cannot easily pass through the blood-brain barrier. Obviously, GAMG can be transferred into the brain through an unknown transport mechanism, and GA cannot enter into the brain. These results indicated that GAMG can be developed as a new drug targeting lung, kidney, live, even brain, exerting multiple physiological activities [5,6,7,8], without adverse effects on cardiovascular and cerebrovascular diseases. All in all, GAMG had more widely tissue distribution than the metabolite GA after intragastric administration, which further supported that GAMG had stronger pharmacological activity.

## 4. Conclusions

The study on the pharmacokinetics and tissue distribution of GAMG was used to understand that GAMG exhibited better biological functions than GL. An HPLC-MS/MS method was developed to simultaneously quantify GAMG and GA in rat plasma and tissues. Analytes were detected by MRM scans in positive-ion mode. The linearity, LLOQ, specificity, recovery rate, matrix effect met the requirements of quantitative analysis. For metabolite GAMG, *C*_max_ (2377.57 ng/mL), *T*_max_ (5 min), and AUC_0-T_ (6625.54 mg/L*h) after oral GAMG were much higher than *C*_max_ (346.03 ng/mL), *T*_max_ (2.00 h), and AUC_0-T_ (459.32 mg/L*h) after oral GL. GAMG can be directly and rapidly absorbed and had excellent bioavailability. For metabolite GA, GAMG had close *C*_max_, *T*_max_ and AUC_0-T_ to GL. After oral administration, the tissue distribution of GAMG was rapid and high in kidney (3.859 μg/g), spleen (3.167 μg/g), small intestine (2.509 μg/g), liver (1.946 μg/g), lung (1.035 μg/g), and brain (1.012 μg/g) at 0.5 h, while that of GA gradually increased to their peaks in lung (0.705 μg/g), kidney (0.479 μg/g) and liver (0.332 μg/g) until 12 h. Physiology-based pharmacokinetic and tissue distribution studies on GAMG suggested that oral GAMG can be rapidly and efficiently absorbed to exert stronger physiological functions and be widely distributed in tissues to exert multiple pharmacological activities. This will provide a reference for the clinical application of GAMG.

## Figures and Tables

**Table 1:** Calibration curves of GAMG in different biological samples.

Biosamples	Linear Range (ng/mL)	Calibration Curves	Correlation Coeffcient (r)	LLOQs (ng/mL)	RSD of LLOQs (*n* = 6, %)	RE of LLOQs (*n* = 6, %)
Plasma	2–300	Y = 0.3823X − 0.0239	0.999	2	3.35	7.50
Heart	10–1000	Y = 1.4309X + 0.4599	0.996	10	3.96	11.00
Liver	10–2000	Y = 1.4784X + 0.3639	0.998	10	5.85	7.23
Spleen	10–2000	Y = 1.0012X + 0.5079	0.999	10	9.11	9.40
Lung	10–1000	Y = 0.9201X + 0.9474	0.997	10	8.30	12.40
Kidney	10–2000	Y = 0.8114X + 0.4544	0.999	10	8.26	12.76
Brain	10–2000	Y = 0.9922X + 0.0052	0.998	10	2.70	5.45
Small Intestine	10–2000	Y = 0.3612X + 0.0062	0.999	10	7.48	12.92

**Table 2:** Calibration curves of GA in different biological samples.

Biosamples	Linear Range (ng/mL)	Calibration Curves	Correlation Coeffcient (r)	LLOQs (ng/mL)	RSD of LLOQs (*n* = 6, %)	RE of LLOQs (*n* = 6, %)
Plasma	0.5–150	Y = 0.9184X + 0.1610	0.998	0.5	14.74	6.96
Heart	5–500	Y = 0.4259X − 0.0296	0.998	5	7.32	13.11
Liver	5–1000	Y = 0.4925X − 0.0781	0.996	5	5.82	11.99
Spleen	1–200	Y = 0.4261X − 0.0052	0.998	1	4.09	8.90
Lung	5–500	Y = 0.2573X + 0.0066	0.996	5	5.34	13.82
Kidney	5–200	Y = 0.2164X + 0.0263	0.996	5	12.20	10.28
Brain	5–200	Y = 0.2201X + 0.0267	0.997	5	4.00	12.51
Small Intestine	1–200	Y = 0.2292X + 0.0723	0.998	1	8.20	11.58

**Table 3:** Intra-assay and inter-assay precision and accuracy of each analyte (*n* = 6).

Analyte	Theoretical Concentration (ng/mL)	Intraday (*n* = 6)			Interday (*n* = 6)		
		Mean Measure Concentration	RSD (%)	RE (%)	Mean Measure Concentration	RSD (%)	RE (%)
GAMG	3	3.31	14.20	10.33	3.08	12.99	2.67
	40	41.26	1.94	3.15	42.70	5.36	6.75
	250	253.74	4.64	1.5	258.99	4.31	3.60
GA	1.5	1.71	13.28	14.00	1.67	11.24	11.33
	20	22.95	10.54	14.75	22.02	12.85	10.10
	120	121.33	6.33	1.11	113.98	8.86	−5.02

**Table 4:** Extraction recovery rate and matrix effect of the detection of each analyte (*n* = 6).

Analyte	Theoretical Concentration (ng/mL)	Sample Extraction Recovery Rate (%, Mean ± SD)	Internal Standard Extraction Recovery Rate (%, Mean ± SD)	Sample Extraction Recovery Rate (%, RSD)	Internal Standard Extraction Recovery Rate (%, RSD)	MF_Sample_% (%, Mean ± SD)	MF_IS_% (%, Mean ± SD)	Matrix Factor Normalized by Internal Standard (%, CV)
GAMG	3	68.94 ± 8.76	97.01 ± 9.05	12.71	9.05	78.12 ± 8.44	75.43 ± 7.43	11.77
	40	73.84 ± 6.92	70.34 ± 7.14	9.37	10.15	126.14 ± 7.55	113.63 ± 15.48	12.91
	250	109.94 ± 8.33	97.62 ± 9.96	7.58	9.96	76.46 ± 7.12	97.40 ± 12.25	7.20
GA	1.5	104.96 ± 11.41	102.12 ± 12.09	10.87	11.84	67.30 ± 6.47	71.59 ± 8.61	6.88
	20	93.07 ± 8.41	70.27 ± 6.13	9.04	11.94	89.36 ± 5.48	113.06 ± 9.07	8.89
	120	119.61 ± 10.49	97.53 ± 8.82	8.77	9.04	83.08 ± 5.56	97.19 ± 9.94	13.60

**Table 5:** The stability of each analyte under different storage conditions (*n* = 3).

Analyte	Theoretical Concentration (ng/mL)	Short-Term Room Temperature Stability at 25 °C, 4 h (%, Mean ± SD)	Short-Term Room Temperature Stability at 25 °C, 4 h (%, RE)	Long-Term Frozen Storage Stability at −40 °C (%, Mean ± SD)	Long-Term Frozen Storage Stability at −40 °C (%, RE)	Repeated Freeze-Thaw Stability (%, Mean ± SD)	Repeated Freeze-thaw Stability (%, RE)
GAMG	3	2.87 ± 0.12	−4.33	2.78 ± 0.06	−7.33	2.73 ± 0.03	−9.00
	40	45.45 ± 0.10	13.62	35.12 ± 0.58	1.66	38.07 ± 0.88	−4.82
	250	251.81 ± 5.15	0.72	212.88 ± 3.84	14.85	228.37 ± 9.46	−8.65
GA	1.5	2.43 ± 0.07	−2.8	2.37 ± 0.14	−5.20	2.41 ± 0.34	−3.60
	20	21.55 ± 0.31	7.55	17.63 ± 0.33	−11.85	18.45 ± 0.28	−7.75
	120	121.23 ± 1.91	1.03	107.62 ± 3.35	3.11	107.62 ± 3.35	−10.32

**Table 6:** The statistical difference of the main PK parameters of GAMG in different dose groups.

Pharmacokinetic Parameters	*p* Value		
	Low Dose Group	Medium Dose Group	High Dose Group
*C*_max_ (ng/mL)	0.874	0.710	0.941
*T*_max_ (h)	1.00	1.00	1.00
*T*_1/2_ (h)	0.605	0.733	0.423
AUC_0-t_ (ng/mL*h)	0.244	0.058	0.960
MRT_0-t_ (h)	0.369	0.474	0.831
*V*_d_ (mL/kg)	0.946	0.377	0.348
CL (mL/h/kg)	0.232	0.083	0.667

**Table 7:** The statistical difference of the main PK parameters of GA in different dose groups.

Pharmacokinetic Parameters	*p* Value		
	Low Dose Group	Medium Dose Group	High Dose Group
*C*_max_ (ng/mL)	0.688	0.229	0.412
*T*_max_ (h)	0.801	0.134	0.391
*T*_1/2_ (h)	0.570	0.777	0.124
AUC_0-t_ (ng/mL*h)	0.244	0.157	0.682
MRT_0-t_ (h)	0.499	0.714	0.508
*V*_d_ (mL/kg)	0.838	0.361	0.302
CL (mL/h/kg)	0.638	0.163	0.493

**Table 8:** Pharmacokinetic parameters of GAMG after intragastric administration of GL and GAMG (*n* = 6).

PK Parameters	GL Group (*n* = 6, Mean ± SD)	GAMG Group (*n* = 6, Mean ± SD)
*C*_max_ (ng/mL)	346.03 ± 145.13	2377.57 ± 547.40
*T*_max_ (h)	2.00 ± 0.00	0.083 ± 0.00
*T*_1/2_ (h)	8.18 ± 2.48	15.73 ± 7.26
AUC_0-T_ (mg/L*h)	459.32 ± 80.81	6625.54 ± 1680.70
MRT_0-T_ (h)	17.54 ± 2.81	11.22 ± 2.58
*V*_d_ (mL/kg)	133.15 ± 41.06	99.25 ± 56.43
CL (mL/h/kg)	11.30 ± 1.93	4.85 ± 1.59

**Table 9:** Pharmacokinetic parameters of GA after intragastric administration of GL and GAMG (*n* = 6).

PK Parameters	GL Group (*n* = 6, Mean ± SD)	GAMG Group (*n* = 6, Mean ± SD)
*C*_max_ (ng/mL)	747.08 ± 236.85	1412.58 ± 80.83
*T*_max_ (h)	13.67 ± 0.82	12.33 ± 0.82
*T*_1/2_ (h)	7.54 ± 2.86	8.48 ± 5.00
AUC_0-T_ (mg/L*h)	11,598.49 ± 4496.08	15,252.54 ± 4661.22
MRT_0-T_ (h)	18.14 ± 2.35	15.99 ± 1.07
*V*_d_ (mL/kg)	32.06 ± 15.70	32.26 ± 36.29
CL (mL/h/kg)	3.00 ± 1.38	2.20 ± 0.94

## Data Availability

Data are contained within the article and Supplementary Materials.

## Supplementary Materials

The following supporting information can be downloaded at: https://www.mdpi.com/article/10.3390/molecules27144657/s1, Figure S1: Total ion current of product ions for each analyte. Figure S2: Specific chromatograms of each component in rat plasma. Figure S3: Linear relationship between GAMG dose and *C*_max_. Figure S4: Linear relationship between GAMG dose and AUC_0-T_. Table S1: Dilute reliable quality control samples. Table S2: Pharmacokinetic parameters of GAMG after intragastric administration of three dose groups of GAMG in rats. Table S3: Pharmacokinetic parameters of GAMG after intragastric administration of three dose groups of GA in rats. Table S4: Concentration distribution results of GAMG and GA in various tissues.
