# Supplementary material for: Physiology-Based Pharmacokinetic Study on 18β-Glycyrrhetic Acid Mono-Glucuronide (GAMG) Prior to Glycyrrhizin in Rats
Source: Molecules. 2022 Jul 21;27(14):4657. doi: 10.3390/molecules27144657 (PMC9315563; doi:10.3390/molecules27144657)
Supplement: Supplementary file 1 [file molecules-27-04657-s001.zip › molecules-1796806-supplementary.pdf]

Physiology-based pharmacokinetic study on 18β-glycyrrhetic acid mono-glucuronide (GAMG) prior to glycyrrhizin in rats

Mengxin Cao [a,b,1], Jiawei Zuo [a,1], Jian-Guo Yang [a], Chenyao Wu [a], Yongan Yang [c], Wenjian Tang [a,*], Lili Zhu [a,*]

[a] School of Pharmacy, Anhui Province Key Laboratory of Major Autoimmune Diseases, Anhui Medical University, Hefei 230032, China

[b] Huainan Municipal Food and Drug Inspection Center, Huainan 232000, China

[c] Jiangsu Nature Biological Engineering Technology Co., Ltd, Nanjing 210023, China

Contents

Figure S1 Total ion current of product ions for each analyte..**S2**

Figure S2 Specific chromatograms of each component in rat plasma. **S3**

Figure S3 Linear relationship between GAMG dose and C_{max}....................................**S4**

Figure S4 Linear relationship between GAMG dose and AUC_{0-T}..................................**S4**

Figure S5 Average plasma concentration-time curve of each analyte **S5**

Table S1 Dilute reliable quality control samples...**S5**

Table S2 Pharmacokinetic parameters of GAMG after intragastric administration of three dose groups of GAMG in rats...**S6**

Table S3 Pharmacokinetic parameters of GAMG after intragastric administration of three dose groups of GA in rats...**S7**

Table S4 Pharmacokinetic parameters of GAMG after ig two groups of rats...............**S8**

Table S5 Pharmacokinetic parameters of GA after two groups of rats..........................**S8**

Table S6 Concentration distribution results of GAMG and GA in various tissues........**S9**

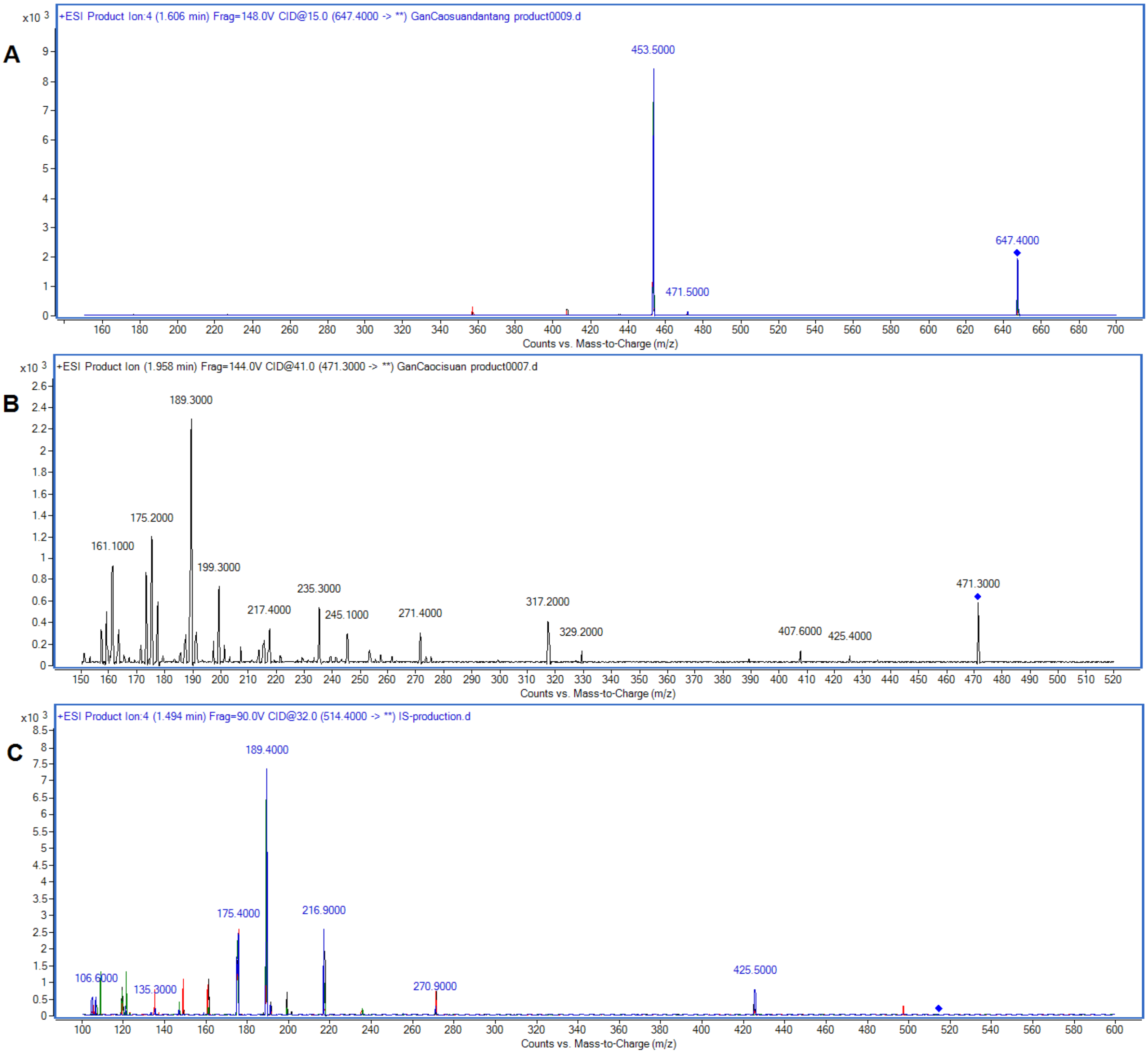

Figure S1. Total ion current of product ions for each analyte (**A**: GAM; **B**: GA; **C**: IS).

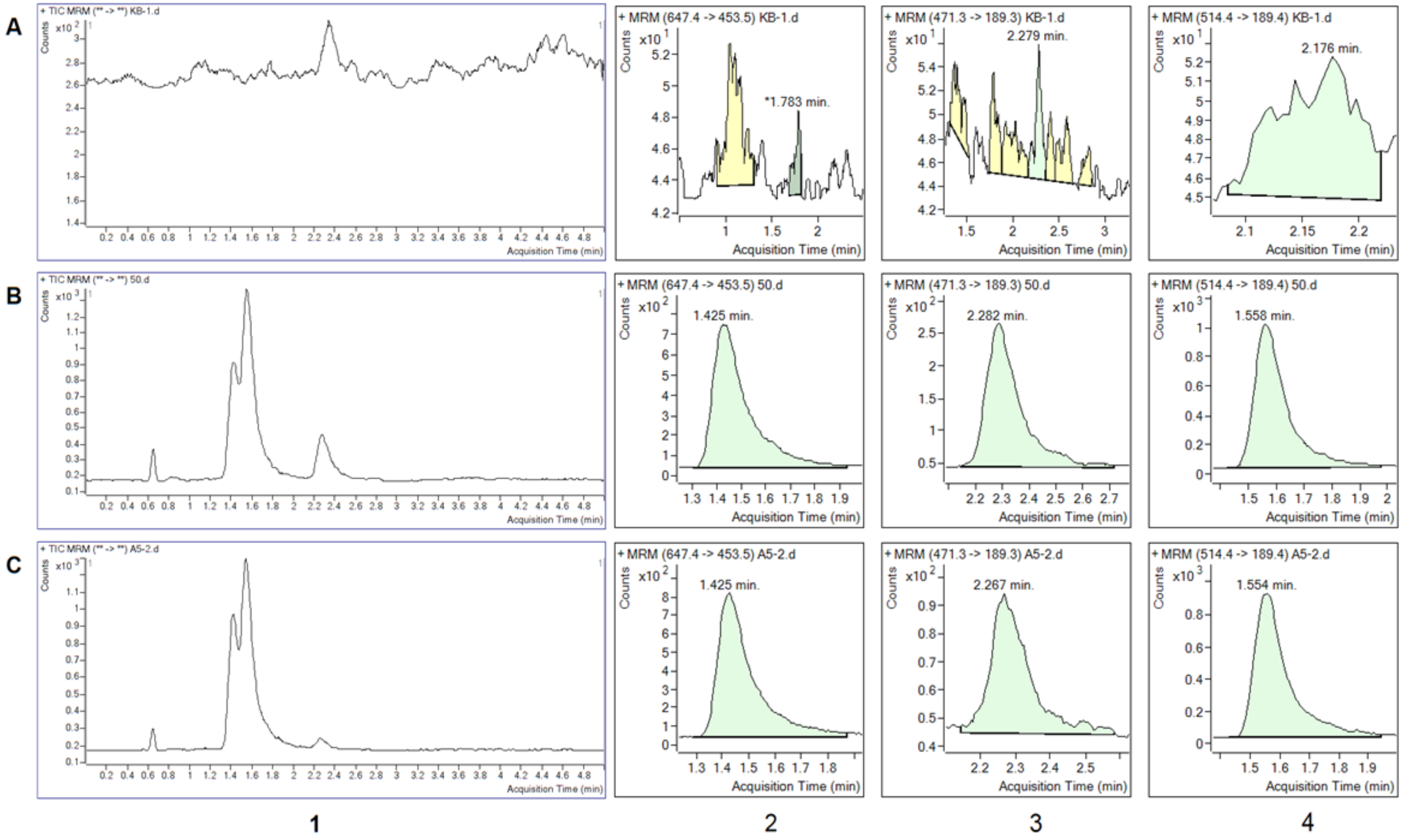

Figure S2. Specific chromatograms of each component in rat plasma. **A**: blank plasma sample; **B**: blank rat plasma added of standard references (GAMG 50 ng/mL, GA 50 ng/mL, IS 50 ng/mL); **C**: rat plasma sample obtained 0.25 h after ig 7.5 mg/kg GAMG. (1: Total ion current diagram; 2: GAMG; 3: GA; 4: IS).

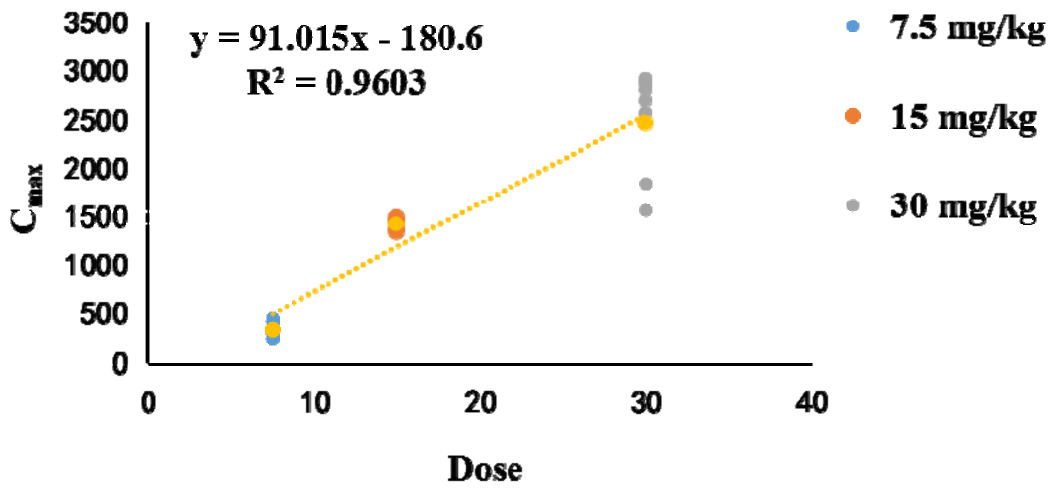

Figure S3. Linear relationship between GAMG dose and C_{max}.

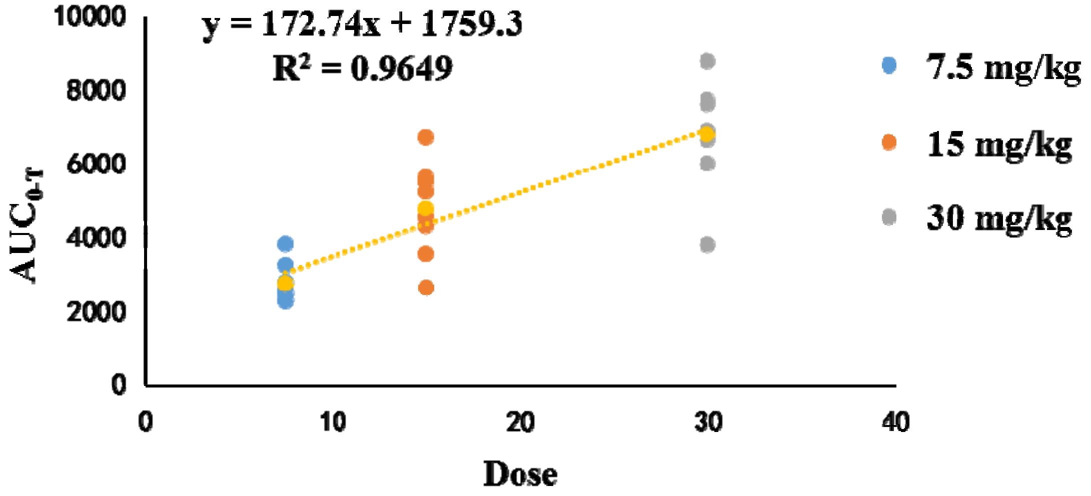

Figure S4. Linear relationship between GAMG dose and $AUC_{0\text{-}T}$.

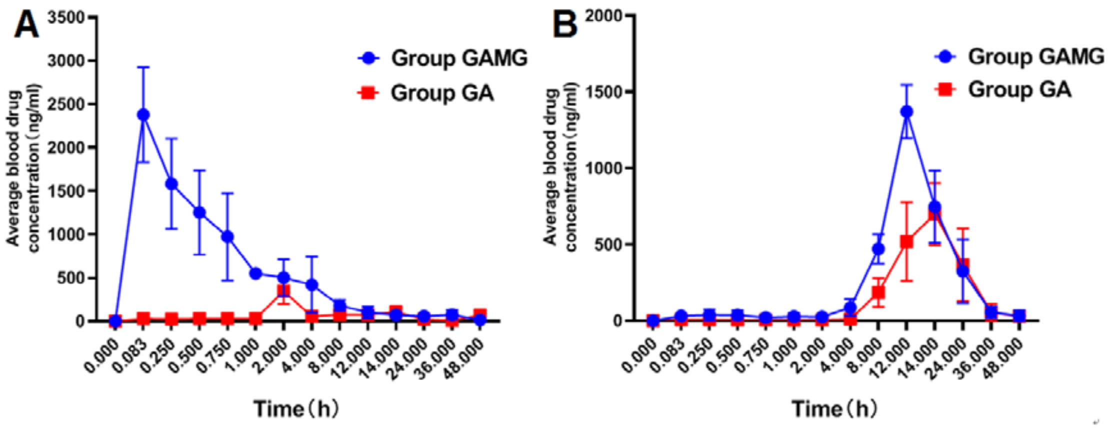

Figure S5. Average plasma concentration-time curve of each analyte (**A**: GAMG; **B**: GA) after ig two groups of rats (n = 6).

Table S1 Dilute reliable quality control samples (n = 5)

Analyte	Theoretical concentration (ng/mL)	Mean measure concentration (ng/mL)	RSD (%)	RE (%)
GAMG	40	40.93	0.85	2.08
	250	246.71	8.41	-1.32
	2500	2556.96	1.44	2.28
GA	40	37.25	7.62	-6.88
	250	238.56	3.84	-4.58
	500	538.94	2.65	7.89

Table S2 Pharmacokinetic parameters of GAMG after intragastric administration of three dose groups of GAMG in rats.

Group	Pharmacokinetic parameters	Female (n = 4, mean ± SD)	Male (n = 4, mean ± SD)	Total (n = 8, mean ± SD)
Low dose group	C_{max} (ng/mL)	336.45 ± 57.93	346.86 ± 111.70	341.65 ± 82.56
	T_{max} (h)	0.083 ± 0.00	0.083 ± 0.00	0.083 ± 0.00
	$T_{1/2}$ (h)	19.73 ± 4.57	17.38 ± 7.32	18.56 ± 5.79
	$AUC_{0\text{-}T}$ (mg/L*h)	2526.84 ± 211.48	3012.39 ± 665.19	2769.61 ± 525.51
	$MRT_{0\text{-}T}$ (h)	17.36 ± 1.92	15.32 ± 3.74	16.34 ± 2.96
	V_d (mL/kg)	92.93 ± 31.06	94.70 ± 40.13	93.82 ± 33.23
	CL (mL/h/kg)	2.98 ± 0.24	2.58 ± 0.55	2.78 ± 0.45
Medium dose group	C_{max} (ng/mL)	1416.09 ± 70.04	1434.21 ± 61.24	1425.15 ± 61.67
	T_{max} (h)	0.083 ± 0.00	0.083 ± 0.00	0.083 ± 0.00
	$T_{1/2}$ (h)	15.30 ± 0.73	16.71 ± 7.87	16.00 ± 5.23
	$AUC_{0\text{-}T}$ (mg/L*h)	5610.41 ± 899.61	3945.85 ± 1103.04	4778.13 ± 1288.38
	$MRT_{0\text{-}T}$ (h)	14.65 ± 5.50	12.31 ± 2.75	13.48 ± 4.22
	V_d (mL/kg)	60.58 ± 12.08	142.67 ± 141.96	101.63 ± 112.96
	CL (mL/h/kg)	2.73 ± 0.44	4.05 ± 1.20	3.39 ± 1.20
High dose group	C_{max} (ng/mL)	2454.84 ± 42.46	2484.49 ± 632.79	2469.66 ± 502.01
	T_{max} (h)	0.083 ± 0.00	0.083 ± 0.00	0.083 ± 0.00
	$T_{1/2}$ (h)	12.73 ± 4.89	16.71 ± 7.87	14.72 ± 6.43
	$AUC_{0\text{-}T}$ (mg/L*h)	6769.46 ± 2133.97	6828.23 ± 713.66	6798.84 ± 1473.40
	$MRT_{0\text{-}T}$ (h)	11.85 ± 3.06	12.31 ± 2.75	12.08 ± 2.70
	V_d (mL/kg)	73.96 ± 33.08	109.72 ± 61.99	91.84 ± 49.81
	CL (mL/h/kg)	4.90 ± 2.04	4.43 ± 0.46	4.67 ± 1.39

Table S3 Pharmacokinetic parameters of GAMG after intragastric administration of three dose groups of GA in rats.

Group	Pharmacokinetic parameters	Female (n = 4, mean ± SD)	Male (n = 4, mean ± SD)	Total (n = 8, mean ± SD)
Low dose group	C_{max} (ng/mL)	352.45 ± 121.84	384.61 ± 91.70	368.53 ± 101.30
	T_{max} (h)	10.00 ± 2.31	10.50 ± 3.00	10.25 ± 2.49
	$T_{1/2}$ (h)	6.10 ± 2.25	7.00 ± 1.96	6.54 ± 2.01
	AUC_{0-T} (mg/L*h)	3951.21 ± 956.06	4327.73 ± 1012.36	4139.47 ± 933.53
	MRT_{0-T} (h)	15.38 ± 2.54	16.40 ± 1.06	15.89 ± 1.88
	V_d (mL/kg)	71.01 ± 37.22	77.13 ± 43.77	74.07 ± 37.76
	CL (mL/h/kg)	7.94 ± 1.90	7.27 ± 1.92	7.60 ± 1.80
Medium dose group	C_{max} (ng/mL)	688.87 ± 90.18	784.98 ± 115.60	736.93 ± 107.06
	T_{max} (h)	10.00 ± 2.31	12.00 ± 0.00	11.00 ± 1.85
	$T_{1/2}$ (h)	6.92 ± 1.04	6.72 ± 0.86	6.82 ± 0.89
	AUC_{0-T} (mg/L*h)	7637.86 ± 1172.14	6342.56 ± 1094.78	6990.21 ± 1257.71
	MRT_{0-T} (h)	13.58 ± 0.96	13.80 ± 0.69	13.69 ± 0.78
	V_d (mL/kg)	40.53 ± 12.22	46.77 ± 3.14	43.65 ± 8.90
	CL (mL/h/kg)	4.00 ± 0.65	4.84 ± 0.62	4.42 ± 0.82
High dose group	C_{max} (ng/mL)	1403.44 ± 98.14	1453.97 ± 59.30	1428.70 ± 79.78
	T_{max} (h)	12.50 ± 1.00	12.00 ± 0.00	12.25 ± 0.71
	$T_{1/2}$ (h)	10.23 ± 5.43	5.36 ± 0.54	7.79 ± 4.42
	AUC_{0-T} (mg/L*h)	14463.37 ± 5415.07	15789.13 ± 2952.52	15126.25 ± 4099.42
	MRT_{0-T} (h)	16.07 ± 1.23	15.56 ± 0.72	15.82 ± 0.97
	V_d (mL/kg)	41.82 ± 42.73	15.22 ± 4.13	28.52 ± 31.50
	CL (mL/h/kg)	2.38 ± 1.13	1.95 ± 0.36	2.17 ± 0.81

Table S4 Pharmacokinetic parameters of GAMG after ig for two groups of rats (n = 6)

PK parameters	GL group (n = 6, mean ± SD)	GAMG group (n = 6, mean ± SD)
C_{max} (ng/mL)	346.03 ± 145.13	2377.57 ± 547.40
T_{max} (h)	2.00 ± 0.00	0.083 ± 0.00
$T_{1/2}$ (h)	8.18 ± 2.48	15.73 ± 7.26
AUC_{0-T} (mg/L*h)	459.32 ± 80.81	6625.54 ± 1680.70
MRT_{0-T} (h)	17.54 ± 2.81	11.22 ± 2.58
V_d (mL/kg)	133.15 ± 41.06	99.25 ± 56.43
CL (mL/h/kg)	11.30 ± 1.93	4.85 ± 1.59

Table S5 Pharmacokinetic parameters of GA after two groups of rats (n = 6)

PK parameters	GL group (n = 6, mean ± SD)	GAMG group (n = 6, mean ± SD)
C_{max} (ng/mL)	747.08 ± 236.85	1412.58 ± 80.83
T_{max} (h)	13.67 ± 0.82	12.33 ± 0.82
$T_{1/2}$ (h)	7.54 ± 2.86	8.48 ± 5.00
AUC_{0-T} (mg/L*h)	11598.49 ± 4496.08	15252.54 ± 4661.22
MRT_{0-T} (h)	18.14 ± 2.35	15.99 ± 1.07
V_d (mL/kg)	32.06 ± 15.70	32.26 ± 36.29
CL (mL/h/kg)	3.00 ± 1.38	2.20 ± 0.94

Table S6 Concentration distribution results of GAMG and GA in various tissues (n = 6)

tissue	Analyte	Con. (μg/g)				
		0	0.5 h	1 h	4 h	12 h
heart	GAMG	0	0.657 ± 0.231	0.135 ± 0.042	0.086 ± 0.023	0.606 ± 0.278
	GA	0	0.049 ± 0.014	0.027 ± 0.004	0.086 ± 0.023	0.158 ± 0.045
liver	GAMG	0	1.946 ± 0.499	0.800 ± 0.291	0.096 ± 0.046	0.245 ± 0.077
	GA	0	0.089 ± 0.013	0.054 ± 0.016	0.142 ± 0.072	0.332 ± 0.060
spleen	GAMG	0	3.167 ± 0.765	0.386 ± 0.111	0.743 ± 0.228	0.894 ± 0.130
	GA	0	0.087 ± 0.039	0.012 ± 0.008	0.018 ± 0.008	0.052 ± 0.019
lung	GAMG	0	1.035 ± 0.083	0.869 ± 0.249	0.436 ± 0.139	1.942 ± 0.227
	GA	0	0.072 ± 0.018	0.027 ± 0.008	0.259 ± 0.069	0.705 ± 0.134
kidney	GAMG	0	3.859 ± 0.537	0.867 ± 0.151	0.271 ± 0.063	1.136 ± 0.153
	GA	0	0.054 ± 0.033	0.014 ± 0.005	0.193 ± 0.085	0.479 ± 0.124
brain	GAMG	0	1.012 ± 0.142	0.481 ± 0.105	0.259 ± 0.099	0.288 ± 0.033
	GA	0	0	0	0	0
Small intestine	GAMG	0	2.509 ± 0.729	5.634 ± 0.165	0.493 ± 0.181	0.164 ± 0.045
	GA	0	0.032 ± 0.029	0.108 ± 0.104	0.070 ± 0.093	0.032 ± 0.023
